# Needs of Internally Displaced Women and Children in Baghdad, Karbala, and Kirkuk, Iraq

**DOI:** 10.1371/currents.dis.fefc1fc62c02ecaedec2c25910442828

**Published:** 2016-06-10

**Authors:** Riyadh Lafta, Nesreen A Aflouk, Saba Dhiaa, Emily Lyles, Gilbert Burnham

**Affiliations:** Al Mustansiriya University, Faculty of Medicine, Department of Community Medicine, Baghdad, Iraq; Al Mustansiriya University, Faculty of Medicine, Department of Community Medicine, Baghdad, Iraq; College of Health and Medical Technology, Department of Community Health, Baghdad, Iraq; College of Health and Medical Technology, Department of Community Health, Baghdad, Iraq; Center for Refugee and Disaster Response, Department of International Health, the Johns Hopkins Bloomberg School of Public Health, Baltimore, MD, USA; College of Health and Medical Technology, Department of Community Health, Baghdad, Iraq; Center for Refugee and Disaster Response, The Johns Hopkins Bloomberg School of Public Health, Baltimore, MD, USA

## Abstract

**Background:**

The continuing conflict in Iraq has now created an estimated four million internally displaced persons (IDPs). The bulk of recently displaced persons are in Central Iraq, often in insecure and difficult situations.

**Objective:**

To determine the health status and health needs of women and children, age 15 and under, among a sample of this IDP population in Kirkuk, Baghdad, and Karbala governorates.

**Methods:**

Data were collected from the senior female in 1216 families which contained 3665 children living in 45 makeshift settlements.

Findings: The majority of IDPs were living in tents or religious centers. Repeated displacements were common. Kidnappings were reported by 5.2% of families, and 7.9% of families reported a death of a family member during or after displacement. Intentional violence accounted for 72.3% of deaths. Only a third of children in school at the time of displacement continued in school. On average, households had received assistance on 3.2 occasions since displacement, food being the most common form. Access to health services was difficult. Some form of transport was often required. Few women knew where to secure antenatal services and many did not know where childhood immunization services were available. During or after displacement 307 women had delivered or were currently pregnant. Complications of pregnancies were common, with a quarter reporting anemia, and 22.1% experiencing hemorrhage. Both communicable and non-communicable diseases (NCDs) were common in the women and children in the survey. Scabies, diarrhea and lice were common among children. Among women, hypertension accounted for 36.6% of NCDs and type 2 diabetes for 15.9%. Domestic violence directed against women was reported in 17.4% of families and against children in 26.6%

**Interpretation:**

Women and children in IDP settlements of Central Iraq experience many vulnerabilities involving their health, education and their environment, in addition to living in physical danger. While some external assistance was received, much more is needed to meet the needs of a displaced population which is unlikely to return home soon.

## Introduction

Iraq currently has about 4 million internally displaced persons (IDPs), 10.8% of its population and 10% of IDPs worldwide.^1^ Major Iraqi population movements started with the March 2003 invasion by coalition forces which created millions of refugees and IDPs. Displacement on a sectarian basis was particularly marked during 2006-2010. Subsequently, as the conflict cooled, IDP numbers dropped, reaching 1.1 million in September 2013^2^.

The Islamic State in Iraq and Syria (ISIS/ISIL^1^) assault into Iraq displaced an estimated 480,000 persons from Al Anbar governorate in the first half of 2014.^3^ During June and July of 2014, a further 505,482 persons were displaced, mostly from Mosul, Iraq’s second city.^4^ ^5^ Starting in August 2014, 728,700 were displaced from the Mt. Sinjar area, thousands more from the Nineveh plain, and further numbers from military attacks in the Saladin area (Figure 1).

**Growth in IDP population.**
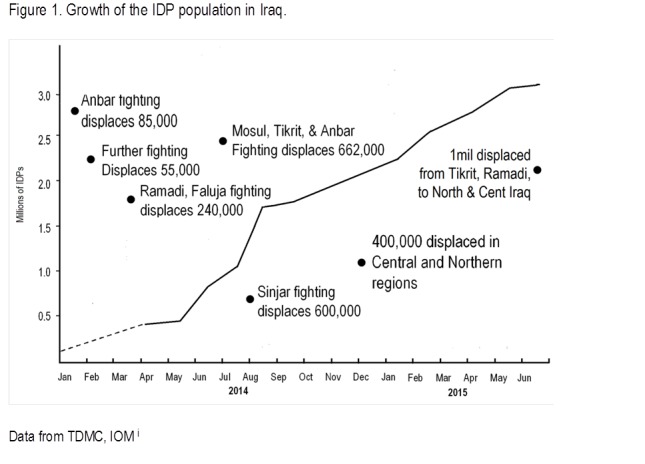

Currently, about 28% of IDPs in Iraq are hosted in the Kurdistan Regional Government (KRG) areas, 68% in central Iraq, and 4% in the South. The governorates hosting the largest IDP populations are Anbar, Baghdad, and Kirkuk.^6^ Some of the displaced live in rented houses or are staying with host families while others live in “critical shelters,” which include informal tented settlements, religious centers, unfinished or abandoned structures, or car parks.^7^ Those living in the critical shelters are among the most vulnerable of the displaced. In the Central area of Iraq there are more IDPs living in these critical shelters than other parts of the country. Only 8% of IDPs in the Central area are housed in organized camps, with an estimated 2.5 million living outside of camps.^8^ ^9^ More than half a million displaced live in Baghdad. Most come from three governorates; (42%) from Al-Anbar governorate, 32% from Nineveh, and 13% from Saladin.

The majority of IDPs are registered with the Ministry of Displacement and Migration or the in the KRG areas with the Department of Displacement and Migration, entitling them to a cash grant of 1 million dinars (US$ 810).^10^ While recent assessments have shown improvement in registration trends, cash assistance has not kept up with the flow of the displaced. The competition for employment among IDPs is intense and the high unemployment affects financial security. The Multi-Cluster Needs Assessment (MNCA II) conducted in October 2015 identified borrowing money to pay rent as one of the major contributors to the large amount of debt among the IDPs, although the survey did not include Baghdad.^11^ As money to pay rent diminishes, IDPs are forced to leave rented apartments for the informal IDP settlement sites which often provide inferior conditions and inadequate access to essential resources and services.

In response to the massive displacements from the ISIS assaults the Inter Agency Standing Committee (IASC) declared Iraq a level 3 (L3) emergency, setting out a “Whole of Iraq” assistance strategy.^12^ With the escalating crisis in 2014, most aid agencies moved their headquarters to the more secure Kurdish areas. Better security there has resulted in more information and greater assistance to IDPs in northern Iraq from aid agencies than for IDPS in central Iraq.^13^ As a consequence, not much is known of the health needs of vulnerable displaced persons in central Iraq. While international agencies may have less presence, local civil society organizations and informal groups have come together spontaneously to provide assistance to IDPs in the central areas. However, without a coordinating body, much IDP information is not uniformly collected or widely shared.

The objective of this study was to obtain further information about the health status and health needs of IDP women and children, aged 15 and under, living in the informal settlements in central Iraq. The study was conducted between February and June 2015.

Our focus was on settlements in Baghdad, Karbala and Kirkuk, which between them hosted an estimated 688,944 IDPs, about a third of Iraq’s IDPs.^14^ This estimate is undoubtedly low as some IDPs were not counted or registered. It is the hope that information from this study will help improve access to services for this vulnerable population. These governorates were chosen as they had been receiving a steady influx of IDPs from the ISIS advances in Anbar, Dyala, Kirkuk, Saladin governorates, and specifically the cities of Ramadi and Mosul. The more southern governorates had received fewer numbers of individuals displaced from ISIS assaults and, as such, were not selected for this study. IDPs in the KRG controlled areas were also not included as their accessibility has meant good data already exist for this group.

## METHODS

The study design was a cross-sectional study conducted during a five month period in early 2015, in Baghdad, Kirkuk and Karbala governorates.

The study participants were women in selected IDP families living in informal settlements who provided information about their health needs and those of their children, aged 15 and under, as well as family characteristics. The desired sample size was about 1000 families. This size was estimated to be sufficient to detect variables of interest with 95% confidence with a power of 80%. Sampling locations are shown in Figure 2.

**Sampling locations. Governorates not to scale.**
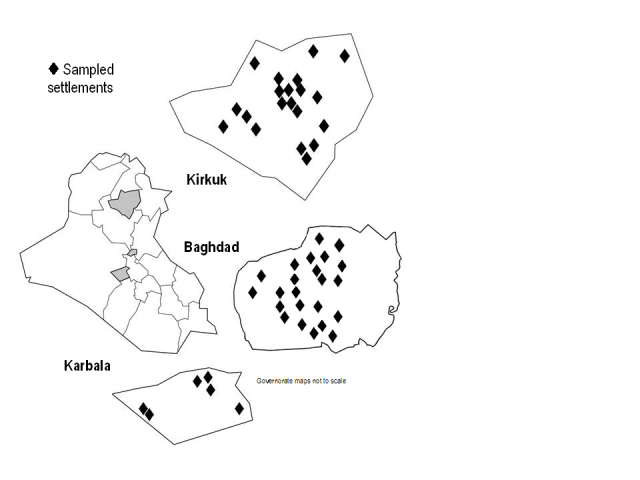

Sampling was based on population data available as of February 2015, including data from an internal report by the Baghdad City Council. Ongoing displacement and lack of registration of many IDPs meant this was most certainly a low estimate.^15^

Sampling was done through a two-stage cluster random sampling technique by taking non-equal clusters from secure and accessible IDP settlements. On entering a settlement, the study team met with the person nominally in charge of the settlement, explaining the nature and purpose of the survey and providing the official letters of approval. Using lists of families maintained by the person in charge of the settlement, a systematic random sample of 5% of families was obtained. A trusted local resident was used as guide to assist in finding the families to be sampled and to inform community members who might have concerns about the survey. Some settlements were very small (fewer than 50 families) and sometimes families were scattered among rented houses or host families, making it difficult to locate a sample of 5% of the settlement population. Where there was a problem sampling from an area because of small, dispersed numbers or security restrictions, the cluster size was increased to 10% of the number of families in accessible and contiguous IDP areas.

On approaching a family, verbal informed consent was obtained from the mother. Where there was more than one woman present, the senior woman was interviewed. The phrasing of questions was sometimes modified as required for a specific cultural or regional background of the woman being interviewed. Sensitive questions concerning pregnancy, emotions and domestic violence were asked very strict confidence and out of hearing of others.

A goal was to draw a sample to adequately represent each settlement population. Some bias in sampling was unavoidable as only a small proportion of IDPs were in settlements, the vast majority being invisibly integrated into the urban environment in unknown locations. Many IDP settlements were under strict security restrictions that excluded access for interviewers. However our sample, taken from informal settlements, is likely from the more vulnerable sector of IDPs, based on information from those providing assistance.

Data were collected using mostly quantitative measures, but with some more qualitative questions. The interviewers collected information about demographics, living circumstances, socioeconomic status, health and social problems after displacement, and access to health care and other services such as school attendance. Semi-structured questions around a few sensitive topics allowed the interviewer to probe for underlying factors, and then group the responses according to her judgement. The questionnaire was written in English, translated into Arabic, and re-translated to English to ensure accuracy. Interviews were conducted by female family medicine specialists with three days of training for this study, and extensive prior experience in community surveys. The interviewers were supervised by the study’s senior researcher.

No personal identifiers were collected. All questions of a sensitive nature were conducted out of ear shot of other persons. Completed questions were kept in a secure location. Computer entered data carried no specific location information. Ethical approval for the survey was obtained from the Iraqi Council for Medical Specialization and the Iraq Ministry of Health and the work carried out in accordance with the WMA Declaration of Helsinki. Permission to conduct the survey was obtained from the Baghdad Provincial Council and appropriate governorate authorities. Analysis of the data was declared exempt by the Institutional Review Board of the Johns Hopkins Bloomberg School of Public Health.

Descriptive statistical analysis was carried out on the full data set using Stata version 13 (College Station, TX). The Stata ‘svy’ command was used to account for the cluster survey design so that standard errors of point estimates and model coefficients were adjusted for survey design effects. Subgroups were compared using chi squared and t-test methods. Simple logistic regression methods were used to obtain odds ratios.

## RESULTS

**Demographic findings.** Data were collected from women representing 1216 families in 45 IDP settlements. This represents or approximately 1% of the total estimated number of 115,000 displaced families in the three included governorates (Table 1).

**Table 1. Location of 1216 families sampled.** *governorates for 66 children missing.

Governorate	Total no. IDP locations	Estimated number of IDP households	Estimated number of IDPs	Families sampled	Children 15 and under*
Baghdad	505	45,607	273,642	521	1612
Kirkuk	95	57,440	344,640	544	514
Karbala	104	11,777	70,662	151	1471
Totals	704	114,824	688,944	1216	3663

In Baghdad, 521 families from 20 settlements were interviewed; in Kirkuk, 544 families from 19 settlements, and in Karbala, 151 families from six settlements (Figure 2). In all, 1216 women were interviewed from families with in total 3663 children. No woman declined to participate. Demographic characteristics of the families interviewed are listed in Table 2.

**Table 2. Demographic characteristics of families of the 1216 women interviewed.** 

	
Number of women interviewed	1216
Median age of women	35.0 years
Median family size	7.0
Total number of children 15 and under	3663
Number of children under age 5	1013 (27.6%)
Median age of all children aged 15 and under	8.0 years
Median number of children per family	3.0 children
Children attending school at the time of displacement	2335
Children currently attending school	789 (33.8%)
Total number of male children aged 15 and under	1910 (52.1%)
Female headed families	215 (17.1%)
Illiterate women	319 (26.2%)
Women completed primary school or able to read and write	690 (56.8%)
Women not working outside the family	1153 (94.8%)
Median number of times family displaced	2.0 (range 1-10)

Much of the survey population was displaced from January 2014 through June 2015, with 662 (54.3%) families being displaced between January and June 2014 (Table 3).

**Table 3. Displacement characteristics for the families of 1216 IDP women interviewed:** 

Number of displacements	No.	(%)
1	497	40.9
2	30	2.4
3	358	29.4
4	207	17.0
5	57	4.6
6	29	2.3
7	21	1.7
8	11	0.9
9 or more	6	0.5
subtotal for more than one displacement	718	
Total displaced	1216	100
Reasons for displacement if more than one displacement	692	96.3
Security	125	17.4
Economic	1	0.1
Home Damaged	818*	113.8*
Date of Displacement		
January - June 2014	662	54.3
July- December 2015	391	32.2
January - June 2015	163	13.5
Total	1216	100
*some women gave more than one reason		
		

In 2015, only 163 (13.5%) of surveyed families had been displaced by the end of May. Of those displaced, 498 (40.9%) were displaced only once, 318 (26.2%) had two or three displacements, and 331 (27.2%) had four or more displacements. IDPs currently resident in Baghdad reported a mean of 3.2 (95% CI =2.7-3.6, p<0.001) displacements, a higher number than for other governorates. Lack of security was cited as the reason for displacement by nearly all (96.3%) families.

**Family circumstances**. The circumstances for IDP living arrangements are detailed in Table 4.

**Table 4. Housing type and services available for the families of 1216 women interviewed.** 

	Number	Percent
Shelter type		
Tent	273	22.5
Religious building	253	20.8
House	188	15.5
Government Institution	119	9.8
Hotel	57	4.7
Camp (Caravan)	28	2.3
School	9	0.7
Others*	289	23.8
Source of water supply		
Tanker trucks	635	52.2
Water mains	581	47.8
Toilets		
Public	936	77.0
Individual or family	280	23.0
Toilet is clean	766	63.0
Cooking fuel		
Gas	919	75.6
Other	104	8.6
None	192	15.8
Electrical power		
Available	990	81.4
Not connected	226	18.6
*including unfinished/abandoned buildings, hotels, caravans, and carparks		

The majority of families were sheltered in tents or religious centers. Crowding was a problem with an average of 6.5 persons per sleeping room or space. Only 417 (34.4%) shelters had fewer than five persons sleeping together at night and only 27% of families had more than one sleeping room. Food supplies were considered adequate by 967 (79.5%) families. Gas was the major source of cooking fuel, but there were 192 (15.8%) families which lacked any cooking facilities. Of the 2335 children who had been in school at the time of displacement, only 789 (33.8%) of these children had continued in school after the initial displacement. Children of mothers who had completed secondary school had the highest odds of continuing in school after displacement (OR 2.9, 95%CI 1.96-4.41, p<0001).

Kidnapping of a family member was reported by 66 (5.2%) families, with seven families having had more than one member taken (Table 5).

**Table 5. Deaths during and after displacement for 1216 families interviewed.** 

	Number	Percent
Members of family kidnapped		
0	1150	94.6
1	59	4.9
2 or more	7	0.6
Total kidnapped	66	5.5
Number of deaths since displacement		
No deaths	1138	93.6
1 death	76	6.3
2 or more deaths	21	1.7
Total deaths	97	7.9
Age of deceased at time of death		
<10 yrs	9	9.3
10-19 years	7	7.2
20-29 years	34	35.1
30-39 years	17	17.5
40 - 49 years	9	9.3
50-59 years	10	10.3
60+ years	11	11.3
Sex of deceased (all ages)		
Male	72	74.2
Female	25	25.8
Cause of death		
Gun shot	61	61.8
Explosion and shells	7	7.3
Beheaded	2	2.1
Breast cancer	2	2.1
Cerebrovascular accident	3	3.1
Car Crash	2	2.1
Heart Disease	14	14.4
Others*	6	4.3
*pneuomnia, renal failure, heat stroke, diarrhea		

Families headed by females more likely to have a family member kidnapping as compared to families with a male head of household (x2 34.6, p<0.001). Kidnappings occurred at a similar level among families fleeing Anbar, Diyala, Mosul, and Saladin (2.7%-6.1%). However, a much higher number (23 or 39.7%) of the 58 families fleeing Kirkuk reported one or more persons kidnapped from their family than from other governorates (x2 93.4, p<0.001)

There were 97 families (7.9%) who reported the death of a member during or after displacement (Table 5).The median age at death among adults was 30.0 years. Three-quarters of these deaths were among males. The median age of death for children was 1.25 years. The leading cause of reported deaths among both adults and children was intentional violence (72.3% of deaths). Among the 57 individuals with intentional causes of death (gun shot, ordnance, beheadings), 45.6% were between 20 and 29 years of age and 87.4% were males. Deaths were not statistically more common for families displaced from a specific governorate or for the number of displacements.

** Economics**. Of women interviewed, 1153 (94.8%) stated they had no current employment outside of the family. A regular salary was the principal source of income reported by 296 (24.3%) families. Other income sources included savings (13.9%), non-governmental organizations (NGOs) (8.8%), and a variety of other sources (48.2%). This latter category included gifts, remittances from abroad, and assistance from local charities. All but 35 (2.9%) of the families in this survey reported having received some form of assistance since displacement.

On average, assistance was reported by families to have been received 3.4 times (95%CI, 3.1-3.8). The principal sources of assistance were NGOs (811 or 68% of families), followed by government assistance (107 or 17.5%), and from individuals or other groups (153 or 13.0%). The most common types of assistance reported were food (reported by 96.9%), clothing (65.4%), household goods (50%), and cash (40.2%). The frequency with which assistance was received was not related to the educational level of the mother, family size, or number of displacements.

**Access to health care.** There were 694 (57.1%) women who indicated that Primary Health Care (PHC) was accessible to the areas of their residence. Access to the services of health visitors (a doctor or nurse) was reported by 338 (27.8%) women, and 162 (13.3%) reported that mobile clinics were regularly available. Only 22 (1.8%) families reported a hospital as their primary access for care. PHC services were accessible on foot to 280 (23.0%) families while the remaining 77% of families indicated that a car or other transport was needed to access these services. When asked about specific services, 708 women (63.3%) knew where children’s immunizations were available, but only 48 (4.0%) women were aware of any antenatal services available.

**Common disease conditions**. Conditions classified as infectious or communicable had affected some 851 (17.4%) women and children since the time of displacement. Scabies was the most common communicable disease among respondents, reported by 305 (35.7%) persons; next was diarrhea (245, 28.7%) and lice infestation (207, 24.2%). Other common communicable conditions reported since displacement included pneumonia in 32 and tuberculosis in seven.

Non-communicable diseases as diagnosed by a health worker were reported by 656 of the women interviewed. Hypertension was reported by 240 (36.6% of those with any NCD), diabetes by 104 (15.9%), asthma by 100 (8.3%), cardiovascular disease by 95 (14.5%), and arthritis by 84 (12.8%). Other conditions include migraine, peptic ulcer, malignancies, and irritable bowel syndrome.

Since displacement, some 430 of the 4879 women and children had been hospitalized for various conditions other than pregnancy. These numbers included 159 children under age five admitted, constituting 37.1% of all admissions reported. Overall, the media age of pediatric admissions was 4.0 years, equally divided between males and females. The average age of adult women admitted to hospital was 41.0 years.

**Maternal health.** Since displacement, there were 307 pregnancies among those women interviewed of which 157 women had delivered a live birth, 41 spontaneous abortions had occurred, and 109 women were currently pregnant.. Of those 157 deliveries 130 (82.8%) deliveries occurred in hospital or health facility. The remaining 27 delivered at their place of residence at the time. Complications of pregnancies were frequently, including anemia in 77 (25.1%), hemorrhage (antepartum or postpartum) in 68 (22.1%) and hypertension in 41 (15.9%).

**Injuries.** During or since displacement injuries were reported for 113 (2.3%) persons, of whom 10 (9%) were children. Hospitalization was required for 53 (47%) of these injuries, and 41 (36%) had a surgical procedure. Surgery was more commonly required for intentional injuries (55.4%) compared with unintentional injuries (19%). The most common sites of injuries were the upper extremities in 40 (35.4%), the head and neck 32 (28.3%), and the lower extremities 25 (22.1%). Falls were associated with 30 injuries (26.4%) and intentional violence (bullets, explosions and shell injuries) were responsible for 53 injuries (46.9%).

**Domestic violence**. There were 211 (17.4%) women interviewed who indicated domestic violence had occurred, directed toward women of the family, and in 317 instances (26.6%), directed toward a child in the family. Physical violence among children was reported as a problem by 419 (34.4%) families. In 44 (3.6%) families a marriage of a girl under 18 had taken place following displacement. Further questions about sexual and gender-based violence were not asked.

**Emotions**. Women were asked in confidence about their feelings and emotions while being displaced. There were 755 (61%) women who reported they felt sad, and 230 (18.9%) who felt panic at times. The odds of reported feelings of panic increased 1.37 (95%CI 1.02-1.81, p=0.031) for each additional displacement. The odds of reporting sadness or panic did not increase with the death of a family member. Other less common feelings included depression, anxiety, and suicidal thoughts. Suicidal attempts were admitted by three women.

## DISCUSSION

These data present a number of serious public health problems among the IDPs of central Iraq. The difficulty in accessing this population by the international agencies, compared with those in the Kurdish region reduces the awareness of needs in central Iraq. The pattern of privation and difficult access to health care which we found in this study is likely repeated many times over among the nearly four million IDPs of Iraq, particularly those in the central region. Families have been uprooted several times in the flight from the advancing ISIS fighters. Family members have been killed or kidnapped while others have died from conditions which may have been treatable by accessible health services in a more stable and environment. Even in settlements, access to health services was low. Translating the findings of this study into action is a major challenge given limited resources and continuing instability.

This study focused on vulnerable families on the margins of Iraqi’s millions of displaced who live in identified settlements. At the same time it provides an important window into the lives of the displaced living in some of the less accessible situations. Similar or worse conditions are likely to exist among families scattered through urban areas living singly in rented quarters, derelict buildings, car parks, or staying with host families.

The greatest challenge to this population is basic day-to-day subsistence. While aid from multiple resources is reaching IDP families, the average distribution of 3.2 aid deliveries since the time of displacement is hardly adequate for many people who had been displaced for one year or more at the time of this study. IDPs are eligible for monthly government cash grants, though these are now becoming increasingly harder to access. In an attempt to improve access to assistance in August 2015, the United Nations (UN) announced the launch of a national humanitarian hotline. Using this Iraqis can receive information about assistance available, request aid, and provide confidential feedback on humanitarian efforts.^6^ This followed a successful hotline pilot in Erbil. In addition to improving accountability of aid organizations, if accompanied by additional specifically targeted education programs, this hotline has the potential to improve knowledge of available resources and overall health and living conditions among IDPs.

Women respondents identified NGOs as the most active in providing their families with assistance. This NGO group would include various international groups such as the World Food Program (WFP), United Nations High Commissioner for Refugees (UNHCR), and International Organization for Migration (IOM) as well as numerous local civil society organizations. In spite of having been displaced for a year or longer, most families reported having received only occasional assistance, most commonly in the form of food. In many cases food was distributed as part of the Iraqi Public Distribution System (PDS), established in 1991. Many IDPs were registered with the PDS at their permanent residence, but their access to food aid was interrupted during the often slow process of having their records transferred to their current location.^13^ Cash was received by some 40% of the surveyed displaced population, which was the most flexible form of assistance. Despite cash, food items and the PDS, a quarter of families still reported inadequate access to food. As international assistance for Iraq dwindles, it is likely that food insecurity will increase. The 15.8% of families reporting no form of cooking fuel, combined with decreasing food assistance, poses further nutritional concerns. Even if IDPs are able to access food, inability to adequately prepare it could increase incidence of food-borne illness and further worsen the population’s nutritional status.

The collapse of oil prices and an increase in military expenditures in Iraq has caused the per capita GDP to contract by some 19% in 2015, and led to surging unemployment.^16^ For the country as a whole, poverty levels increased to 22.5% in 2014, with the number of people living below the poverty line increased by an estimated 2.8 million. Among the displaced there is a fear of leaving camps to look for work, lest they be perceived as suspected ISIS supporters and physically attacked.^13^ Though a few of the women in this study had some form of employment, any income earned by employed women would hardly be enough to support families. Family support came from a combination of gifts, savings, casual labor, and trading, as well as through assistance from friends and family, few of which sources are sustainable in the long-term.

The effects of conflict on the schooling of children across Iraq have also been a great concern, with an estimated 2 million children missing school in the past year.^17^ In the vulnerable population reported here, only one-third of children previously in school prior to displacement were currently attending school. UNICEF and other agencies are addressing this problem, though challenges are numerous and these extend to finding the space for additional schools and locating the teachers, who themselves have fled.

The IDPs in our study reported that access to primary health care services was not particularly easy, with only 57.1% of women stating that PHC services were readily available. Even where these services were available, only 23.0% indicated that these could be reached on foot. At the same time, the presence of Health Visitors was reported to be available to about a quarter of women interviewed. The community access program by qualified health workers is an established program in some other parts of Iraq. Antenatal services were known to be available by only 4.0% of women interviewed. This is of particular concern, as the accounts from the 307 women who were pregnant or who had delivered during or after displacement showed that many serious and avoidable complications had occurred. Pregnant displace women are at high risk, as they often left their homes suddenly, sometimes under violent circumstances, and then experienced further displacements under duress. These stressful circumstances could certainly have a negative effect on their pregnancy. Insufficient food could lead to anemia and micronutrient deficiencies in pregnancy. Women may be forced to give birth in unsanitary and/or unsafe conditions, which may lack access to emergency obstetrical care, further increasing risks of life-threatening complications.

Similarly of concern is that only 63% of surveyed women knew where EPI vaccination services could be obtained. In the first half of 2015, Iraq experienced 1352 measles cases, the majority of which were in Baghdad and adjacent Babil, raising serious concerns about the apparent limited knowledge of where to receive vaccinations.^18^ Limited knowledge of where to access vaccinations may also suggest that mothers were uncertain where other child health services were available. Diarrhea, commonly reported by mothers at all sites, was particularly prevalent among those living in settlements or caravans. Expanding resources for treating children’s diarrhea and improving knowledge of such services available to IDPs would reduce the number of diarrheal cases. Identifying the most common causes of diarrhea outbreaks and improving water and sanitation in settlements could lessen diarrhea in IDP children in these particularly vulnerable environments.

All families reported access to water. Sources of water were about evenly divided between water mains and supplies from tanker trucks. Piped water is unreliable in urban areas of Iraq. Many IDP tented sites were supplied from tanks containing chlorinated water brought in by truck. As a primary source water, at times IDPs in some locations purchased water from vendors. Where water mains exist they are often in a badly deteriorated state. In the November 2015 outbreak, the largest number of cholera cases were in Baghdad and Babil. This was thought to be associated with the heavy rains of October 2015, which caused many areas in Baghdad to be flooded with raw sewage.^19^ ^20^ Most latrine were public latrines which generally are more difficult to keep clean than private or family latrines, and could add to the risk. However, the general cleanliness of latrines, both private and public, was judged to be good in the majority of sites where the interviewers inspected them.

Among family of the women interviewed, non-communicable diseases were common. This is consistent with the relatively high prevalence of non-communicable diseases seen in the region among other displaced populations such as Syrian refugees.^21^ The most commonly reported non-communicable diseases were hypertension and type 2 diabetes, both of which are more difficult to manage at the PHC clinic level than are common communicable diseases. Where access to basic primary health care services is difficult, it is likely that access to more specialized care needed for management of many NCDs would be even more difficult.

Deaths were common during the time of flight or while living in temporary settlements. Among interviewed families, 97 (7.9%) reported death of at least one member since displacement. The violent circumstances associated with the IDP displacement was reflected by 70 (72.2%) of these deaths were due to intentional violence. Given the nature of conflict in Iraq with high velocity weapons, the deliberate targeting of civilians, and the multiple displacements of this population, this figure is not surprising.

A survey of this nature has many limitations. A sampling frame of all IDPs in Central Iraq was not available. Even if it had been, the numbers would be constantly changing as new displacements occurred and families moved in and out of settlements. As the numbers of IDPs in settlements was in constant flux, we cannot be sure that precisely the same proportions of families were sampled in each settlement. There are likely to be many other vulnerable families, out of sight in abandoned or partially constructed buildings whose circumstances may have been even more vulnerable. IDP families living with hosts are likely to be vulnerable in different ways from those we found in settlements, and they were unreached by this survey. The lack of a secure environment around many settlement means that some IDP families could not be reached, and their condition could be substantially worse than those in relatively more secure situations. Although all interviewers were female doctors and experienced in surveys, IDP women may have been reticent to answer sensitive questions. We limited this survey to families, but in displaced populations the household may be a much larger unit, including unrelated persons joining during displacement. By surveying the household instead, the amount of trauma and kidnapping, as well as other violent events may have been larger.

The future situation for IDPs in central Iraq is very uncertain. The border areas with ISIS are unstable with almost certain prospects for continuing violent conflict, and perhaps further population displacement. The probability of a quick return home for IDPs is unlikely and when it does comes it may be extremely slow, given the unpredictable and destructive nature of continuing conflict in Iraq. When return eventually comes, the social fabric, the institutions of the state, and many of livelihoods that sustained these communities for decades cannot be easily reconstructed. For many families, these should be their most productive years, but instead they have been torn apart, traumatized, and now their lives are in indefinite holding patterns. Both families and the country will suffer from this loss.

## Conclusions

The vulnerability of this population is great, and the emotional trauma of multiple displacements, kidnapping and deaths from intentional violence is great. While some aid is reaching families, much more is needed. Though Iraq is a middle income country, reaching the IDPs in central Iraq will take much more in international assistance than is currently being received. Unfortunately, at this time of great need, assistance is being cut back throughout the region because of lack of funding.^10^ The local civil society organizations which have sprung up in many locations to assist IDPs, offer an avenue for targeting international donor assistance. We hope these data will encourage Iraqi government, local NGO organizations and the international aid agencies to strengthen their assistance. IDPs are less visible and draw less attention than refugees, of which there are many in the Middle East region, but with needs of IDPs are as great or greater.

## Competing Interest Statement

The authors have declared that no competing interests exist.

## References

[1] IDMC. Iraq IDP Figures Analysis.

[2] UNHCR. Iraq Factsheet. Quarter 3 2013

[3] UN Assistance Mission for Iraq (UNAMI). Iraq: New IDPs Map by Province (as of 18 June 2014). June 2014.

[4] IOM. IOM Humanitarian Compendium

[5] The Assessment Capacities Project (ACAPS). Iraq Displacement Profile. July 2014.

[6] USAID. Iraq Complex Emergency Fact Sheet. Number 8 Sept 30 2015.

[7] REACH. Multi-cluster needs assessment of internally displaced persons outside of camps. October 2015.

[8] OCHA. Iraq: Humanitarian Crisis, Situation report No. 62 (16-29 September 2015).

[9] OCHA. Iraq Humanitarian Needs Overview, 2015.

[10] IDMC. Iraqi IDPs caught between a rock and a hard place as displacement crisis deepens

[11] REACH. Multi-cluster needs assessment of internally displaced persons outside of camps. October 2015.

[12] United Nations Iraq. UN Declares a ‘Level 3 Emergency’ for Iraq to Ensure More Effective Humanitarian Response. August 2014

[13] Refugees International. Little Aid and Few Options, November 2015.

[14] IOM. Displacement in Iraq reaches nearly 3.2 million.

[15] International Organization for Migration (IOM). Iraq IDP crisis IOM Displacement Tracking Matrix (DTM) Dashboard. February 2015.

[16] World Bank. Iraq economic overviews.

[17] A World at School. Conflict in Iraq leaves two million out of school.

[18] WHO. Iraq: Measles in 2015 (Reporting period January to August, 2015.

[19] CIDRAP. The outbreak of cholera in Iraq has reached 4,858.

[20] OCHA. Flooding wreaks havoc in Iraqi capital. Humanitarian Bulletin 16 October-5 November 2015.

[21] Doocy S, Lyles E, Roberton T, Akhu-Zaheya L, Oweis A, Burnham G. Prevalence and care-seeking for chronic diseases among Syrian refugees in Jordan. BMC Public Health. 2015;15:1097. doi:10.1186/s12889-015-2429-3. 10.1186/s12889-015-2429-3 PMC4628338 26521231

